# Stimuli-Responsive Dual Cross-Linked *N*-Carboxyethylchitosan Hydrogels with Tunable Dissolution Rate

**DOI:** 10.3390/gels7040188

**Published:** 2021-10-29

**Authors:** Svetlana Bratskaya, Anna Skatova, Yuliya Privar, Andrey Boroda, Ekaterina Kantemirova, Mariya Maiorova, Alexander Pestov

**Affiliations:** 1 Institute of Chemistry, Far Eastern Branch of Russian Academy of Sciences, 159, prosp.100-letiya Vladivostoka, 690022 Vladivostok, Russia; militca2@mail.ru (A.S.); privar.juliya@gmail.com (Y.P.); evr2211@gmail.com (E.K.); 2 A.V. Zhirmunsky National Scientific Center of Marine Biology, Far Eastern Branch of Russian Academy of Sciences, 17, Palchevskogo Street, 690041 Vladivostok, Russia; borodandy@gmail.com (A.B.); maiorovamariya@gmail.com (M.M.); 3 I. Ya. Postovsky Institute of Organic Synthesis, Ural Branch of the Russian Academy of Sciences, 20, Sofia Kovalevskoy Street, 620990 Yekaterinburg, Russia; pestov@ios.uran.ru

**Keywords:** hydrogel, dynamic covalent bond, benzoic imine bond, chitosan, carboxyalkylchitosan, salicylaldehyde, glutaraldehyde, cytotoxicity, human dermal fibroblasts, HCT 116

## Abstract

Here, we discuss the applicability of (methylenebis(salicylaldehyde)—MbSA) for the fabrication of the stimuli-responsive *N*-carboxyethylchitosan (CEC) hydrogels with a tunable dissolution rate under physiological conditions. In comparison with non-covalent salicylimine hydrogels, MbSA cross-linking via covalent bis(‘imine clip’) and non-covalent hydrophobic interactions allowed the fabrication of hydrogels with storage moduli > 1 kPa at ten-fold lower aldehyde/CEC molar ratio with the preservation of pH- and amino-acid responsive behavior. Although MbSA-cross-linked CEC hydrogels were stable at neutral and weakly alkaline pH, their disassembly in cell growth medium (Dulbecco’s modified Eagle’s medium, DMEM) under physiological conditions was feasible due to transimination reaction with amino acids contained in DMEM. Depending on the cross-linking density, the complete dissolution time of the fabricated hydrogels varied from 28 h to 11 days. The cytotoxicity of MbSA cross-linked CEC hydrogels toward a human colon carcinoma cell line (HCT 116) and primary human dermal fibroblasts (HDF) was remarkably lower in comparison with CEC-salicylimine hydrogels. Fast gelation, relatively low cytotoxicity, and tunable stimuli-induced disassembly under physiological conditions make MbSA cross-linked CEC hydrogels promising for drug encapsulation and release, 3D printing, cell culturing, and other biomedical applications.

## 1. Introduction

Several decades stimuli-responsive hydrogels have been attracting high interest for the versatile biomedical applications including wound healing, hemostasis, drug delivery, cell encapsulation and release, 3D bioprinting, tissue engineering, etc. [1,2,3]. More recently, the introduction of dynamic covalent chemistry approaches to hydrogels fabrication allowed the development of a wide range of materials, which benefit from the reversibility of physically cross-linked hydrogels and the mechanical stability of the covalently cross-linked ones [4,5,6].

pH-responsive hydrogels with dynamic benzoic imine covalent bonds represent one of the most important systems for the controlled release of bioactive compounds. Such hydrogels for biomedical applications are preferably based on Schiff bases of biocompatible and biodegradable polymers having inherent or chemically introduced amino groups, e.g., proteins, chitosan, and cellulose derivatives [7,8,9,10,11,12]. The pH value of benzoic imine bond cleavage and the hydrogel disassembly rate in such systems can be turned by the selection of a suitable aldehyde/aminopolymer pair [7,13] and CHO/NH_2_ molar ratio [8,10,11].

Interactions of chitosan with different monoaldehydes have been actively studied as a promising strategy for the development of pH-responsive hydrogels [8,10,14], films [9,11,15], and emulsions [13]. However, due to chitosan’s solubility in acidic media only, the fabrication of chitosan-based dynamic supramolecular assemblies under physiological pH is not feasible. To overcome this limitation, hydrogels of water-soluble chitosan derivatives have been proposed based on glycol chitosan and benzaldehyde-capped PEO-PPO-PEO [16], and carboxyalkyl chitosans with salicylaldehyde [17], vanillin [18], dibenzaldehyde-terminated poly(ethylene glycol) [19], and dextran-graft-aniline tetramer-graft-4-formylbenzoic acid [20]. It was also demonstrated that higher stability of benzoic imine bond at physiological pH resulted in a higher storage modulus of the hydrogels of salicylimines of carboxyalkylchitosans in comparison with chitosan-based hydrogels [17]. This is an important issue for biomedical applications due to the known toxicity of aldehydes.

Numerous studies on the antimicrobial properties of chitosan Schiff bases dealt with derivatives with modification degrees from 1 to 12% [21,22] or with very low concentration of highly substituted derivatives [23] to avoid cytotoxic effect of aldehydes on mammal cells. Glutaraldehyde, which is often used as a cross-linker for chitosan scaffolds fabrication, was cytotoxic when aldehyde content was above 10 mol% [24]. However, in most of the reported up to now chitosan-based dynamers with cleavable benzoic imine bonds, high aldehyde grafting density (above 20 mol%) was required to assure not only pH-triggered disassembly but good mechanical properties (storage modulus > 1 kPa) of the hydrogels [8,10,11,18,19,20].

Since gelation in solutions of Schiff bases of chitosan and its derivatives with aromatic monoaldehydes occurs via non-covalent hydrophobic interactions and hydrogen bonding [8,14,17,25], we expected that chemical cross-linking via the formation of bis-Schiff bases would yield stronger hydrogels with preserved stimuli-responsive behavior at lower CHO/NH_2_ molar ratios.

Here, we discuss the applicability of a cross-linker (methylenebis(salicylaldehyde)—MbSA), which is widely used in the synthesis of Schiff-base ligands and chemical sensors, for the fabrication of pH- and amino acid-responsive carboxyalkylchitosan hydrogels with bis(‘imine clip’) chemistry providing controlled disassembly of the hydrogels under physiological conditions.

## 2. Results and Discussion

### 2.1. Rheological Properties of N-(2-Carboxyethyl)chitosan (CEC) Hydrogels with Bis(‘Imine Clip’)

*N*-(2-carboxyethyl)chitosan (CEC) hydrogels with bis(‘imine clip’) were fabricated via cross-linking with methylenebis(salicylaldehyde) (MbSA) at several MbSA/polymer molar ratios; then, their rheological properties were compared with those of the hydrogels of CEC-salicylimine and CEC cross-linked with glutaraldehyde to reveal contributions of hydrophobic interactions and dialdehyde cross-linking to the stability of supramolecular structures. Recently, we have shown that the advantage of carboxyalkylchitosan over chitosan for the fabrication of salicylimine-based stimuli-responsive hydrogels resulted not only from the solubility of derivatives under physiological pH itself but from significantly enhanced hydrophobic ordering in hydrogels of carboxyalkylchitosan-salicylimines due to the higher stability of the dynamic benzoic imine bond at neutral and weakly alkaline media. Taking this into account, we briefly comment below on chitosan hydrogels and focus on CEC hydrogels cross-linked with MbSA due to their high potential for controlled disassembly at physiological pH.

**Figure 1:**
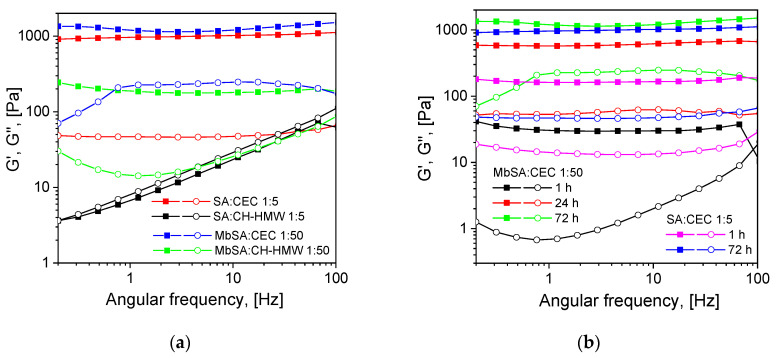
Mechanical spectra of 3% solutions of high molecular weight chitosan (CH-HMW) and *N*-(2-carboxyethyl)chitosan *(*CEC) after the addition of salicylaldehyde (SA) and methylenebis(salicylaldehyde) (MbSA) at SA and MbSA molar ratios to polymers of 1:5 and 1:50, respectively: CEC and CH-HMW solutions, gelation time of 72 h, T = 25 °C (**a**); CEC solution, gelation time of 1 h, 24 h, or 72 h, T = 25 °C (**b**). In both panels: squares—storage moduli (G′), circles—loss moduli (G″).

A comparison of the rheological properties of chitosan and CEC hydrogels formed after the addition of aromatic monoaldehyde (salicylaldehyde—SA) and dialdehyde (MbSA) to polymer solutions revealed the following features (Figure 1 and Table S1, Supplementary Information): (i) MbSA, as a cross-linker capable of forming bis-Schiff bases with primary amino groups of CEC, yields hydrogels with the same storage modulus as can be obtained with SA at a five-fold higher CHO/NH_2_ molar ratio (Figure 1a, taking into account that 1 mole of MbSA contains 2 moles of CHO groups); (ii) hydrogels formed after the addition of both SA and MbSA in weakly alkaline media (CEC-based hydrogels) were remarkably stronger than hydrogels formed in weakly acidic media (chitosan-based hydrogels) at the same CHO/NH_2_ molar ratio (Figure 1a); (iii) the storage moduli of CEC/MbSA hydrogels slowly increased within several days as it was earlier observed for salicylimines of CEC and chitosan [8,17] (Figure 1b).

Thus, rheological data show that chemical cross-linking via bis(‘imine clip’) restricts the dynamics of exchange of a benzoic moiety between adjacent monomer units, so even chitosan hydrogels can be obtained at a very low CHO/NH_2_ molar ratio that was not feasible only via non-covalent interactions in salicylimine–chitosan solutions. However, due to the higher reversibility of the imine bond in acidic media, chitosan hydrogels cross-linked with MbSA at pH 4.5 were much weaker than CEC-based hydrogels formed at pH 8.2. This fact together with the slow evolution of the hydrogels storage moduli suggests that non-covalent interactions, which are the driving force for gelation in salicylimine-based hydrogels, still play an important role in hydrogels chemically cross-linked with MbSA.

**Figure 2:**
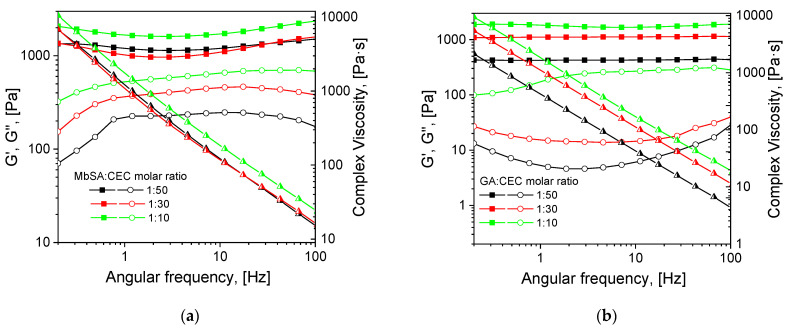
Mechanical spectra of 3% solutions of *N*-(2-carboxyethyl)chitosan (CEC) in 72 h after the addition of cross-linkers at cross-linker/polymer molar ratios of 1:10, 1:30, and 1:50 (T = 25 °C): methylenebis(salicylaldehyde) (MbSA) (**a**); glutaraldehyde (GA) (**b**). In both panels: squares—storage moduli (G′), circles—loss moduli (G″), triangles—complex viscosity.

The role of non-covalent interactions in the gelation of MbSA cross-linked hydrogels became also evident from comparison of their rheological properties with those of CEC hydrogels cross-linked with aliphatic dialdehyde—glutaraldehyde (GA). Figure 2 shows that the storage moduli of CEC hydrogels cross-linked with MbSA and GA are very close at a high cross-linker/CEC molar ratio (1:10), while at a low cross-linking density (molar ratio of 1:50), MbSA yields notably stronger hydrogels. This can be related to the contribution of non-covalent interactions to the stabilization of the supramolecular structure of MbSA-cross-linked hydrogels. As we have recently shown [17], gelation in CEC-salicylimine solution due to non-covalent interactions only was observed already at SA/CEC molar ratio of 1:10. The difference in the evolution of mechanical spectra of CEC hydrogels cross-linked with MbSA (Figure 1b) and GA (Figure S1, Supplementary Information) confirms the completeness of the hydrogel formation via chemical cross-linking with GA within 30 min in contrast to the increase in strength of the MbSA cross-linked hydrogel within several days.

A significant increase in the elastic modulus of salicylimine-chitosan hydrogels, when the temperature was raised from 20 to 40 °C, was earlier observed for non-covalent hydrogels only at a high cross-linking density (at an NH_2_:CHO molar ratio of 2:1) and explained by the shifting of the carbonyl/imine equilibrium to the products at higher temperature [8]. Here, due to the high contribution of bis(‘imine clip’) to the stability of hydrogels, the storage moduli of hydrogels formed at 37 °C were not much higher than those fabricated at 25 °C, but the gelation time was notably faster (Figure S1, Table S1, Supplementary Information).

In comparison with the earlier reported cross-linking of glycol chitosan with benzaldehyde-capped PEO-PPO-PEO [16], MbSA yields hydrogels at a lower molar fraction of cross-linker that correlates with the higher stability of an imine bond formed by aromatic aldehydes with a hydroxyl group in the ortho-position (‘imine clip’). The formation of an ‘imine clip’ between salicylaldehyde and CEC was proved earlier using FT-IR and ^13^C-NMR spectroscopy [17], so the same chemistry can be assumed for cross-linking with MbSA. Despite the low MbSA content in the hydrogels and significant overlapping of MbSA and CEC absorption bands in FT-IR spectra, notable changes were detected in the region of primary and secondary amine absorption (815–910 cm^−1^). The completeness of interactions is confirmed by the absence in the reaction product of a band at 1649 cm^−1^ corresponding to C=O stretching in MbSA and the shift of the band from 1642 cm^−1^ in CEC to 1626 cm^−1^ due to the formation of a new imine bond (Figure S2, Supplementary Information).

### 2.2. Stimuli-Induced Solubility of N-(2-Carboxyethyl)chitosan (CEC) Hydrogels with Bis(‘Imine Clip’)

Due to the strong dependence of benzoic imine bond stability on pH and its higher susceptibility to acidic hydrolysis, pH shift was used to induce the disassembly of Schiff-base hydrogels [8,13,16] at pH < 7 for pH-triggered drug release in gastric site or tumor cells, i.e., in the environments with low pH. However, many materials for biomedical applications, including scaffolds for cell growth and tissue regeneration as well as films and sponges for wound healing, are expected to show stimuli-induced dissolution or resorption under physiological pH. Recently, we have shown that amino acids can work as efficient chemical stimulus for the disassembly of carboxyalkylchitosan-salicylimines hydrogels under physiological conditions [17]. Here, we have investigated how chemical cross-linking affects pH- and amino acids-triggered disassembly of the CEC hydrogels with benzoic imine bonds.

**Figure 3:**
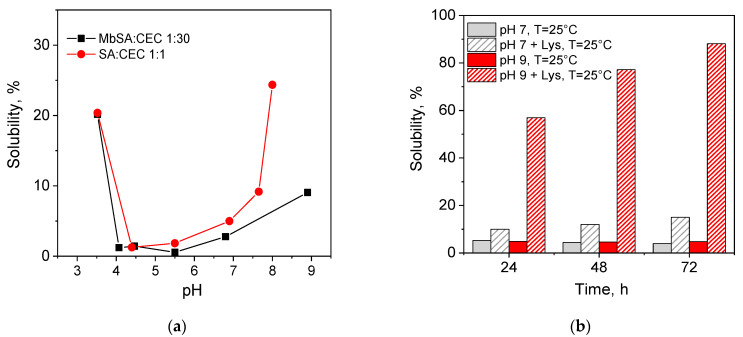
Solubility of *N*-(2-carboxyethyl)chitosan *(*CEC) hydrogels formed via grafting salicylaldehyde (SA) and cross-linking with methylenebis(salicylaldehyde) (MbSA) at molar ratios of 1:1 and 1:30, respectively (**a**). Solubility of MbSA:CEC 1:10 hydrogel in PBS buffer with and without lysine (Lys) addition, lysine concentration 20 g/L (**b**). Dissolution time was 24 h, T = 25 °C, hydrogel/PBS *w*/*w* ratio of 1:10.

Since the mechanism of pH-induced hydrogel disassembly is based on the hydrolysis of a C=N bond, the solubility of both types of CEC hydrogels followed the same trend and increased in acidic and alkaline media (Figure 3a). However, in weakly alkaline and basic media, where the C=N bond is reversible, CEC hydrogel with covalent cross-linkage, bis(‘imine clip’), demonstrated much higher stability in comparison with CEC-salicylimine hydrogel formed via non-covalent interactions only. While in CEC-salicylimine hydrogels the hydrolysis of a C=N bond results in the release of SA and fast loss of hydrophobic ordering at pH > 7.3, in MbSA-cross-linked hydrogels, the movement of the benzoic moieties after the destruction of one ‘imine clip’ was still restricted, and non-covalent interactions stabilized the supramolecular structure. The farther was the pH value from the pH window of high stability for the benzoic C=N bond, the higher was the possibility of simultaneous destruction of both ‘imine clips’ and the lower was the difference in stability of the two types of hydrogels.

**Figure 4:**
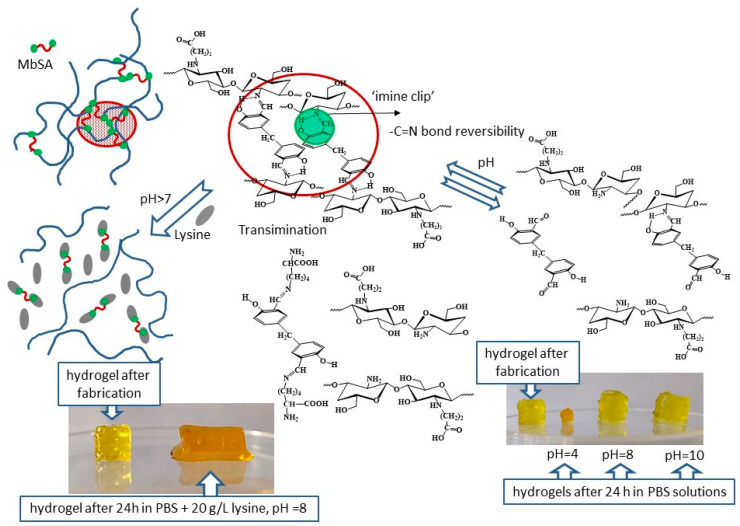
Scheme of pH-and lysine-induced disassembly of *N*-(2-carboxyethyl)chitosan *(*CEC) hydrogels cross-linked with MbSA. Photos correspond to original MbSA/CEC 1:50 hydrogel (gelation time was 72 h) and hydrogel after dissolution in PBS buffers with and without lysine addition at a *w*/*v* ratio of 1:10 and T = 25 °C.

For the same reasons, lysine-triggered disassembly, reported earlier for the CEC-salicylimine hydrogels [17], was also observed for MbSA cross-linked hydrogels (Figure 3b) but at a significantly lower rate. Formation of the lysine Schiff base via a transimination reaction between MbSA:CEC 1:10 hydrogel and lysine (Figure 4) was confirmed by the emergence of the new absorption peak at ≈403 nm in UV-vis spectra and the increase in its intensity during hydrogel dissolution (Figure S3, Supplementary Information). It is important to mention that the solubility values shown in Figure 3 were calculated from the colloid titration data, which allowed quantification of the free polymer fraction released from the hydrogel to the solution [17]. A comparison of the solubility values calculated from colloid titration and weight loss of the CEC hydrogels cross-linked with MbSA at various MbSA:CEC molar ratios showed that in PBS solution (pH 7.4) hydrogels contracted within 1 h and lost up to 80% of their wet weight within 24 h, while only less than 5% of the polymer was released to the solution from all hydrogels during 72 h of observation (Figure S4, Supplementary Information).

In all earlier studies known to us, e.g., [8,13,16], dissolution of the chitosan-based hydrogels with a dynamic imine bond was investigated in PBS solutions. However, in biomedical applications, hydrogels are brought into direct contact either with biological fluids, or cell growth media containing amino acids and proteins, which can significantly affect hydrogels solubility via transimination (Figure 2b and Figure 4). Taking this into account, we have investigated the solubility of CEC hydrogels cross-linked with MbSA in one of the most common cell growth media, DMEM with fetal bovine serum (composition of the media is given in Table S2, Supplementary Information).

**Figure 5:**
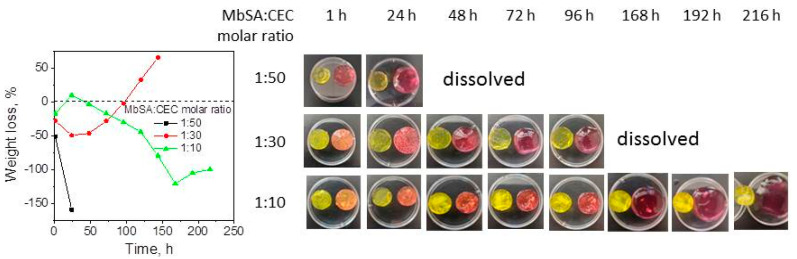
Dissolution of *N*-(2-carboxyethyl)chitosan *(*CEC) hydrogels, cross-linked with methylenebis(salicylaldehyde) (MbSA) at molar ratios MbSA/CEC of 1:10, 1:30, and 1:50 in DMEM at a *w*/*v* ratio of 1:10 and T = 37 °C. Images in each box are given for original hydrogel (**left**) and swollen in DMEM (**right**). Negative values of the weight loss correspond to the hydrogels swelling.

Figure 5 shows that the kinetics of the CEC:MbSA hydrogels weight loss in DMEM significantly depended on the cross-linking density. Weakly cross-linked hydrogels MbSA/CEC at a molar ratio of 1:50 showed in DMEM very fast swelling (Figure 5) and complete dissolution within 28 h. Hydrogel with the highest cross-linking density (MbSA:CEC 1:10) showed at the initial stage a small contraction similar to that observed for hydrogel in PBS buffer, which was followed by slow swelling and complete dissolution in 11 days. At an intermediate cross-linking density (MbSA:CEC 1:30), hydrogel swelling and dissolution also occurred simultaneously and resulted in complete hydrogel disassembly in 7 days. Although the total concentration of amino acids in DMEM was significantly lower (1.606 mg/L or 10.68 mM) than the lysine concentration used in preliminary experiments (Figure 3b), the stability of hydrogels differed drastically in comparison with that in PBS buffer at the same pH value, where only moderate swelling of the hydrogel was observed (Figure 4). This allows the conclusion that CEC hydrogels cross-linked with MbSA can be potentially interesting for the tunable release of encapsulated cells and drugs.

### 2.3. Cytotoxicity of N-(2-Carboxyethyl)chitosan (CEC) Hydrogels

Since both glutaraldehyde, used for the fabrication of chemically cross-linked hydrogels, and salicylaldehyde, used for the fabrication of non-covalent stimuli-responsive hydrogels show relatively high toxicity (LD50 of glutaraldehyde is 134 mg/kg [26] and of salicylaldehyde is 520 mg/kg [27]), stimuli-responsive CEC hydrogels covalently cross-linked with MbSA are expected to have lower toxicity due to the possibility to obtain strong hydrogels at lower aldehyde content.

**Figure 6:**
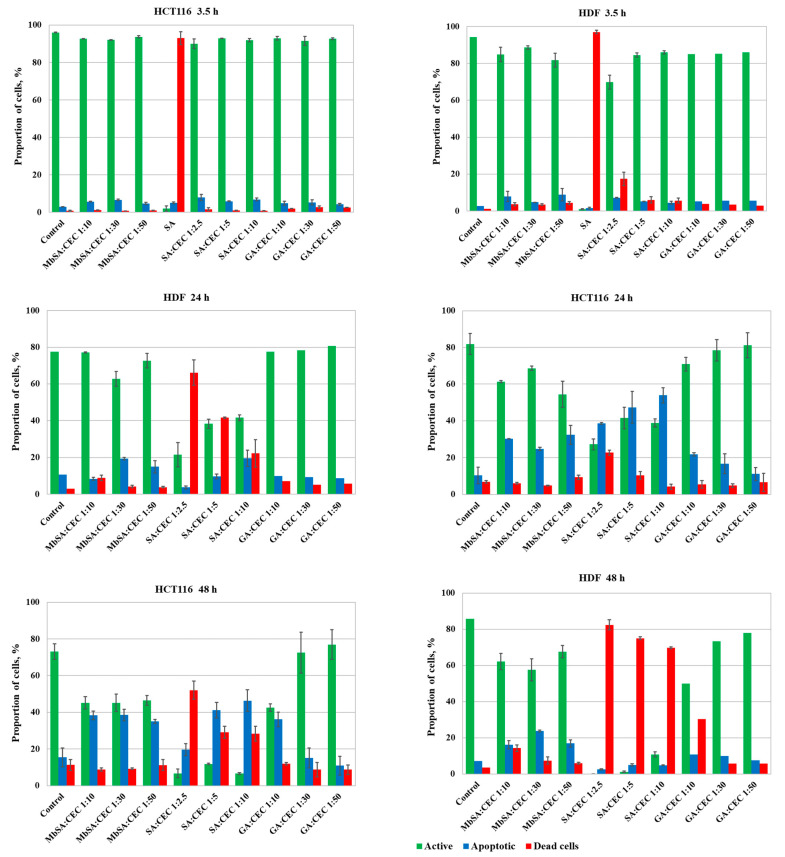
The results of flow cytometrical analysis of human colon carcinoma cell line (HCT 116) and primary human dermal fibroblasts (HDF) cultivated in the presence of hydrogels for 3.5, 24, and 48 h. Cytotoxicity of SA at a concentration corresponding to that in SA:CEC 1:5 hydrogel is given for comparison. The cells were stained with H_2_DCFDA to assess the mitochondrial activity, TO-PRO-3™ to detect apoptotic cells, and DAPI to stain dead cells.

Figure 6 presents a time course of the induced alterations in functional (mitochondrial) activity, proportion of apoptotic and dead human colon carcinoma cells (HCT 116), and primary human dermal fibroblasts (HDF) after incubation with hydrogels. HCT 116 cells were chosen for cytotoxicity tests because of their high capacity to resist different chemicals and even anticancer drugs [28] as well as interest in the formation of 3D tumor models in hydrogels for anticancer drugs screening [29]. HDFs, being a primary cell culture, are relatively sensitive, simple to obtain, and widely used for regenerative medicine purposes and testing toxicants [30,31] as a model of physiologically normal cells.

The proportion of active cells in both control cultures was about 94–96% at the start of incubation and smoothly decreased to 75–85% after 24–48 h, which was probably due to the overgrowth and suppression of cells by other cells. Control cells were adherent to the bottom of polystyrene plate wells with HCT 116 having an epithelial morphology, while HDF has a spindle-shaped fibroblast morphology (Figure S5).

SA at the concentration used for the hydrogel (SA/CEC molar ratio of 1:5) fabrication was highly cytotoxic and resulted in the death of almost all cells (94% of HCT 116 cells and 97% of HDF) even within 3.5 h. Thus, we did not define cell viability after 24 and 48 h incubation with SA. The toxicity of MbSA could not be determined using the same approach, since the precipitates that formed due to the low solubility of MbSA in cell growth medium (DMEM) interfered with cytometry measurements and made microscopic observations impossible.

The incubation of cells with hydrogels for 3.5 h induced a non-significant decrease of active HCT 116 cells to 90–92% and a more pronounced decrease of active HDF to 69–85%. The most drastic changes in the proportion of active cells (and consequent increase of dead cells) were observed after incubation with salicylimine-hydrogels.

Further exposure of cells to salicylimine-hydrogels resulted in a decrease of cellular activity of both cultures to lower than 40% after 24 h with cells detaching from the well bottom and having a round-shape morphology (Figure S5c,d). Then, 48 h incubation with these hydrogels led to the death or apoptosis of more than 90% cells of both cultures. Thus, non-covalent salicylimine-hydrogels, which show good mechanical properties only at high SA grafting density, could not be recommended for prolonged cell culturing.

MbSA- and glutaraldehyde (GA)-cross-linked hydrogels are characterized by lower cytotoxicity, decreasing the proportion of active cells to 60–80% after 24 h of incubation and to about 45–60% with MbSA-cross-linked hydrogels, or about 45–75% with GA-cross-linked hydrogels after 48 h. Most cells remained attached and kept their normal morphology. Moreover, the results revealed the dependency of cytotoxic effects on the GA/CEC molar ratio (the toxicity of hydrogels increased with a cross-linking density), while MbSA-cross-linked hydrogels showed almost the same cytotoxicity for each culture and exposure time. Therefore, aside from providing stimuli-responsive behavior, cross-linking with MbSA allowed the fabrication of stronger hydrogels with lower cytotoxicity in comparison with GA-cross-linked hydrogels.

## 3. Conclusions

Here, we have reported the fabrication of stimuli-responsive hydrogels based on carboxyalkylchitosan derivative cross-linked with methylenebis(salicylaldehyde) via dynamic benzoic imine bonds. We have shown that in comparison with hydrogels formed with salicylaldehyde, as an aromatic monoaldehyde, the formation of a bis(‘imine clip’) provides faster gelation and better mechanical properties of hydrogels at much lower CHO/NH_2_ molar ratio without a loss of stimuli-responsiveness under physiological pH. The higher stability of the ‘imine clip’ over a dynamic imine bond formed by benzaldehyde is responsible for a slower disassembly rate in comparison with the earlier reported chitosan-based hydrogels and makes it possible to vary the dissolution time in cell growth medium from several hours to several days.

The lower cytotoxicity of methylenebis(salicylaldehyde)-cross-linked hydrogels over non-covalent salicylimine-based hydrogels demonstrated that using a human colon carcinoma cell line and primary human dermal fibroblast culture is important for the versatile biomedical applications including wound healing, hemostasis, drug delivery, cell encapsulation and release, 3D bioprinting, and tissue engineering.

## 4. Materials and Methods

### 4.1. Hydrogels Fabrication

Low molecular weight chitosan was purchased from BioLog Heppe GmbH (Landsberg, Germany), the degree of acetylation (DA) was determined by ^1^H NMR spectroscopy to be 0.9, and the viscosity-average molecular weight was 30 kDa. *N*-(2-carboxyethyl)chitosan (CEC) with a carboxyethylation degree of 0.49 was synthesized from CH-LMW as described earlier [32]. High molecular weight chitosan (CH-HMW) with a DA of 0.88 and a molecular weight of ≈5 × 10^5^ Da was purchased from JSC “Bioprogress” (Shchelkovo, Biocombinat, Russia). Salicylaldehyde (SA) of 98% purity was purchased from Sigma-Aldrich (St. Louis, MO, USA).

Methylenebis(salicylaldhyde) (MbSA) was synthesized by treating salicylaldehyde (SA) with formaldehyde using the modified method described in [27]. MbSA yield was 83%, and a purity of 99% was determined by ^1^H NMR spectroscopy (Figure S6, Supplementary Information).

Salicylimine-chitosan (SA:CH-HMW) and salicylimine-CEC (SA:CEC) hydrogels were obtained as follows: salicylaldehyde (SA) purchased from Sigma-Aldrich (98%) was added to 3% solutions of CH-HMW in 1.5% acetic acid (pH adjusted to 4.9) and 3% solutions of CEC in water (pH adjusted to 8.3 with NaOH solution) at an SA/polymer molar ratio of 1:5 (the molar ratios were calculated for the amino group of chitosan, for CEC hydrogel, the same weight amount of SA was used) under constant stirring at 25 °C. CEC hydrogels cross-linked with glutaraldehyde (GA) and methylenebis(salicylaldhyde) (MbSA) were prepared at cross-linker/polymer molar ratios of 1:10, 1:30, and 1:50 by the addition of 5% of GA solution in water or 2% solution of MbSA in DMSO to the 3% polymer solution with pH 8.3 under stirring. Gelation was stopped after 72 h for all types of further investigations except for the monitoring of rheological properties.

### 4.2. Rheological Measurements

The rheological properties of the hydrogels were investigated by recording frequency sweeps in the range between 0.2 and 100 Hz at a temperature of 25 °C or 37 °C and a constant strain of 5% (which was within the linear viscoelastic region) using a Physica MCR 301 rheometer (Anton Paar GmbH, Graz, Austria) with a plate–plate measuring system with a diameter of 25 mm.

### 4.3. Hydrogels Solubility

Hydrogels’ solubility was investigated at 25 °C in PBS buffer (PanEco Ldt., Moscow, Russia); the pH in acidic and basic range was adjusted with H_3_PO_4_ and NaOH, respectively. The solubility experiments were performed as follows: 300 mg of the hydrogel was immersed in 3 mL of PBS solution and gently agitated for 24 h using a Biosan PSU-20i orbital shaker (Latvia) at 30 rpm, and then, an aliquot of the supernatant was taken for colloid titration. Colloid titration was performed using a PC-controlled system connecting a Mütek PCD-04 particle charge detector and a Mütek PCD-T3 Titrator (BTG Instruments GmbH, Weßling, Germany) with 0.001 mol/L standard solutions of polydiallyldimethyl ammonium chloride (PDADMAC) at pH 10.5. CEC solution was titrated using the same procedure after dilution to a concentration of 1 g/L. The concentration of the dissolved polymers calculated from the titration data were expressed in mol of charged groups per L. The hydrogels solubility was calculated using the following formula:

(1) $$Solubility=\frac{CT_{gel}}{CT_{CEC}\times C_{gel}},\;where\;C_{gel}=\frac{\;m_{gel}\times C_{CEC\left(gel\right)}}{\;V_{PBS}\times 100}$$

where *CT_gel_* and *CT_CEC_* are the CEC concentrations (mol/L) in the supernatant after hydrogel dissolution and in CEC solution (1 g/L), respectively; *m_gel_* is the hydrogel weight (g), *C_CEC_*_(*gel*)_ is the concentration of the CEC in hydrogel (%), and *V_PBS_* is the PBS volume used for the hydrogel dissolution (L).

The solubility of MbSA/CEC hydrogels in cell growth media DMEM at 37 °C (see description of the DMEM composition in Section 4.4) was estimated using the gravimetric method, since the presence of protein in DMEM interferes with determination of the solubility using colloid titration. Since the dissolution and swelling of the hydrogels with a dynamic covalent bond occur simultaneously, the positive value of the weight loss corresponded to the domination of dissolution over the swelling, while the negative value—to the hydrogel swelling until complete dissolution.

### 4.4. Hydrogels Cytotoxicity

About 80 milligrams of each hydrogel was placed in each well of a 24-well culture plate (TPP, Trasadingen, Switzerland). The HCT116 cell line (Sigma-Aldrich Corp., St. Louis, MI, USA) was seeded at a density 100 × 10^3^ cells/well in 1 mL of Dulbecco’s modified Eagle’s medium (DMEM, #12800017, Gibco™, Thermo Fisher Scientific, Altrincham, UK) (the complete composition is presented in Table S2, Supplementary Information) supplemented with 10% (*v*/*v*) fetal bovine serum (FBS, HyClone, Logan, UT, USA), 3.7 mg/mL sodium bicarbonate (Sigma-Aldrich), 1× mixture of non-essential amino acids (MEM NEAA, Gibco), 100 U/mL penicillin (Gibco), and 100 µg/mL streptomycin (Gibco). The samples were cultivated at +37 °C, 5% CO_2_, and 90% relative humidity.

After 24 h, the wells were washed with 1 mL of Dulbecco’s phosphate buffer saline (DPBS, Sigma-Aldrich) without Ca^2+^ and Mg^2+^. The solution of 0.05% (*w*/*v*) trypsin—0.02% (*w*/*v*) EDTA was used to detach cells from the wells. A pellet of trypsinized cells from a single well of a 24-well plate was centrifuged at 500× *g* for 5 min and re-suspended in 100 µL of DPBS with 10 µM 2′,7′-dichlorodihydrofluorescein diacetate (H_2_DCFDA) (Sigma-Aldrich) to assess the mitochondrial activity, 1 µM TO-PRO-3™ (Invitrogen, Waltham, MA, USA) to detect apoptotic cells, and 1 µg/mL DAPI (GERBU Biotechnik GmbH, Heidelberg, Germany) to stain dead cells. The cell suspension was incubated in the dark at room temperature for 10 min and then diluted with 150 µL of DPBS. Flow cytometric analyses were conducted within 20 min after staining using a CytoFLEX flow cytometer (Beckman-Coulter, Brea, CA, USA) connected to a computer running CytExpert software (version 2.4, Beckman-Coulter). Single events were determined by a triangle gating on FSC-A against a FSC-H plot in order to exclude cell aggregates from the following analysis. Cells were separated from debris and gel fragments by gating on FSC-A against an FSC-W plot. At least 25,000 cells were evaluated for each sample at a sample flow rate of 60 µL/min, 500–800 events/sec. DAPI was detected at 405 nm excitation and 450/45 BP emission. H_2_DCFDA was detected at 488 nm excitation and 525/40 BP emission. TO-PRO-3™ was detected at 638 nm excitation and 660/10 BP emission. The percentage of dead (DAPI-positive), active (DAPI-negative and H_2_DCFDA-positive), and apoptotic (DAPI-negative and TO-PRO-3™-positive) cells were determined in each sample.

## Data Availability

Data available from the authors upon request.

## Supplementary Materials

The following are available online at https://www.mdpi.com/article/10.3390/gels7040188/s1, Figure S1: Evolution of the mechanical spectra of CEC solution (3%, pH 8.3) after the addition of glutaraldehyde (GA) at T = 25 °C (a) and MbSA at T = 37 °C (b), molar ratio cross-linker: CEC was 1:50: filled symbols—storage modulus (G′), open symbols—loss modulus (G″), half-open symbols—complex viscosity. Figure S2: FT-IR spectra of methylenebis(salicylaldehyde) (MbSA), *N*-(2-carboxyethyl)chitosan (CEC), and lyophilized hydrogels, formed via CEC cross-linking with MbSA at an MbSA/CEC molar ratio of 1:30. Figure S3: Electronic spectra of lysine (Lys) solution and of supernatant after dissolution of MbSA/CEC 1:10 hydrogel in PBS buffer containing 20 g/L of lysine at a hydrogel/PBS *w*/*v* ratio of 1:10, dissolution time was 24 h and 48 h, T = 25 °C. Spectra were recorded using a UV-2600PC scanning UV-vis spectrophotometer (Shimadzu, Kyoto, Japan) in a quartz cuvette with an optical length of 1 cm, spectra of supernatant solutions were recorded after 6.4-times dilution with PBS. Figure S4: Solubility of *N*-(2-carboxyethyl)chitosan *(*CEC) hydrogels, formed via cross-linking with methylenebis(salicylaldehyde) (MbSA) at MbSA/CEC molar ratios of 1:10, 1:30, and 1:50, in PBS buffer, pH = 7.4 determined by the gravimetric method (Weight) and colloid titration (CT). The gravimetric method was used for the swollen hydrogel, so the weight change results from both swelling and dissolution. The dissolution time was 24 h, T = 25 °C or 37 °C, the hydrogel/PBS *w*/*w* ratio was 1:10. Figure S5: The morphology of a human colon carcinoma cell line (HCT 116) (a,c,e,g) and primary human dermal fibroblasts (HDF) (b,d,f,h) cultivated in the presence of gels for 3.5, 24, and 48 h: control cells (a,b), and cells cultured with SA/CEC 1:5 (c,d), GA/CEC 1:50 (e,f), MbSA/CEC 1:50 (g,h). Specimens were examined in a CKX41 inverted microscope (Olympus, Japan) equipped with phase-contrast optics and imaged with an Axiocam 105 color digital camera (Carl Zeiss, Jena, Germany) controlled by ZEN 2 blue edition software (Carl Zeiss). Scale bar—100 µm. Figure S6. 400 MHz ^1^H NMR spectra (DMSO *d*_6_) of methylenebis(salicylaldehyde). Table S1: Rheological parameters of the CEC-hydrogels at angular frequency of 11.2 Hz (full mechanical spectra are shown in Figure 1 and Figure 2). Table S2: The complete composition of Dulbecco’s modified Eagle’s medium.
