# Supplementary material for: Stimuli-Responsive Dual Cross-Linked N-Carboxyethylchitosan Hydrogels with Tunable Dissolution Rate
Source: Gels. 2021 Oct 29;7(4):188. doi: 10.3390/gels7040188 (PMC8628705; doi:10.3390/gels7040188)
Supplement: Supplementary file 1 [file gels-07-00188-s001.zip › gels-1424212-supplementary.pdf]

Supplementary Information to the manuscript:

Stimuli-responsive dual cross-linked N-carboxyethylchitosan hydrogels with tunable dissolution rate

Svetlana Bratskaya [1]*, Anna Skatova [1], Yuliya Privar [1], Andrey Boroda [2], Ekaterina Kantemirova[1], Mariya Maiorova[2], and Alexandr Pestov [3]

1 Institute of Chemistry, Far Eastern Branch of Russian Academy of Sciences, 159, prosp.100-letiya Vladivostoka, 690022 Vladivostok, Russia; sbratska@ich.dvo.ru
2 A.V. Zhirmunsky National Scientific Center of Marine Biology, Far Eastern Branch of Russian Academy of Sciences, 17, Palchevskogo street, 690041 Vladivostok, Russia; borodandy@gmail.com
3 I. Ya. Postovsky Institute of Organic Synthesis, Ural Branch of the Russian Academy of Sciences, 20, S. Kovalevskoy str., Yekaterinburg 620990, Russia; pestov@ios.uran.ru

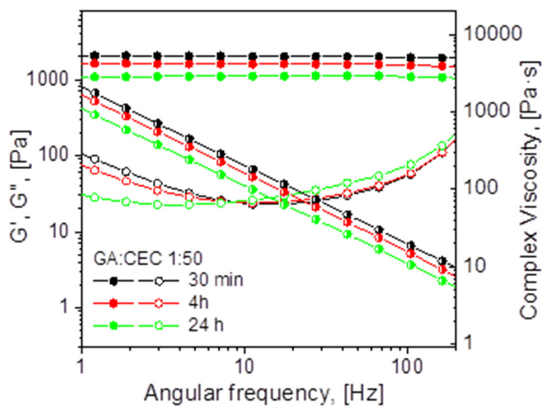

(**a**) (**b**)

Figure S1. Evolution of the mechanical spectra of CEC solution (3%, pH 8.3) after addition of glutaraldehyde (GA) at T=25°C (a) and MbSA at T=37°C (b), molar ratio cross-linker : CEC was 1:50: filled symbols – storage modulus (G'), open symbols – loss modulus (G''), half-open symbols – complex viscosity.

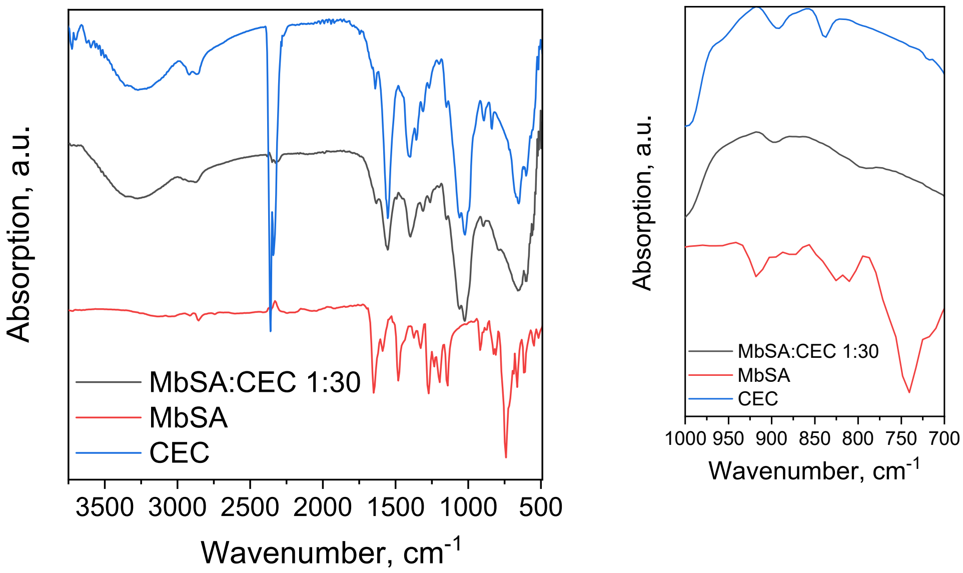

Figure S2. FT-IR spectra of methylenebis(salicylaldehyde) (MbSA), N-(2-carboxyethyl)chitosan (CEC), and lyophilized hydrogels, formed via CEC cross-linking with MbSA at MbSA:CEC molar ratio of 1:30. Fourier transform infrared (FT-IR) spectra were recorded using an IR Affinity-1 spectrometer with a MIRacle 10 FTIR accessory (Shimadzu, Japan).

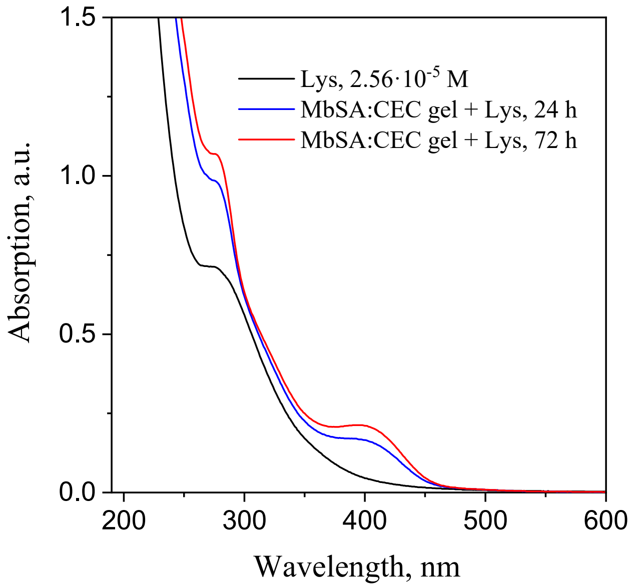

Figure S3. Electronic spectra of lysine (Lys) solution and of supernatant after dissolution of MbSA:CEC 1:10 hydrogel in PBS buffer containing 20 g/L of lysine at hydrogel:PBS w/v ratio of 1:10, dissolution time was 24 h and 48 h, T=25 °C. Spectra were recorded using a UV-2600PC scanning UV-vis spectrophotometer (Shimadzu, Japan) in quartz cuvette with optical length of 1 cm, spectra of supernatant solutions were recorded after 6.4-time dilution with PBS.

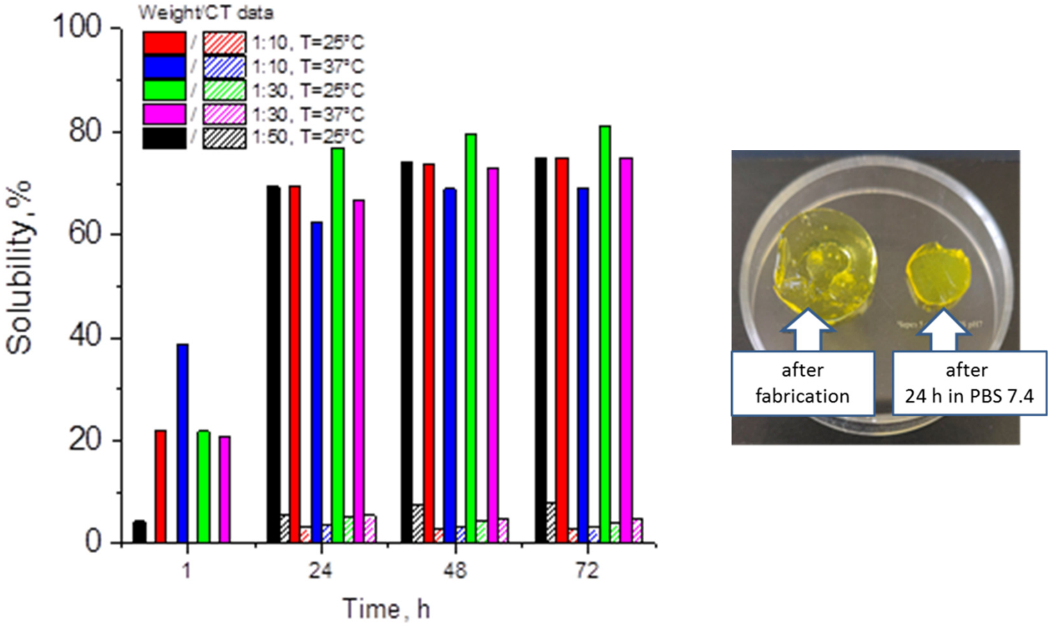

Figure S4. Solubility of N-(2-carboxyethyl)chitosan (CEC) hydrogels, formed via cross-linking with methylenebis(salicylaldehyde) (MbSA) at MbSA:CEC molar ratios of 1:10, 1:30, and 1:50, in PBS buffer, pH =7.4 determined by gravimetric method (Weight) and colloid titration (CT). Gravimetric method was used for the swollen hydrogel, so the weight change results from both swelling and dissolution. Dissolution time was 24 h, T= 25°C or 37°C, hydrogel:PBS w/w ratio was 1:10. Photo:- MbSA:CEC 1:50 hydrogel after fabrication (gelation time was 72 h) and after 24 h in PBS buffer, pH 7.4, T=25°C.

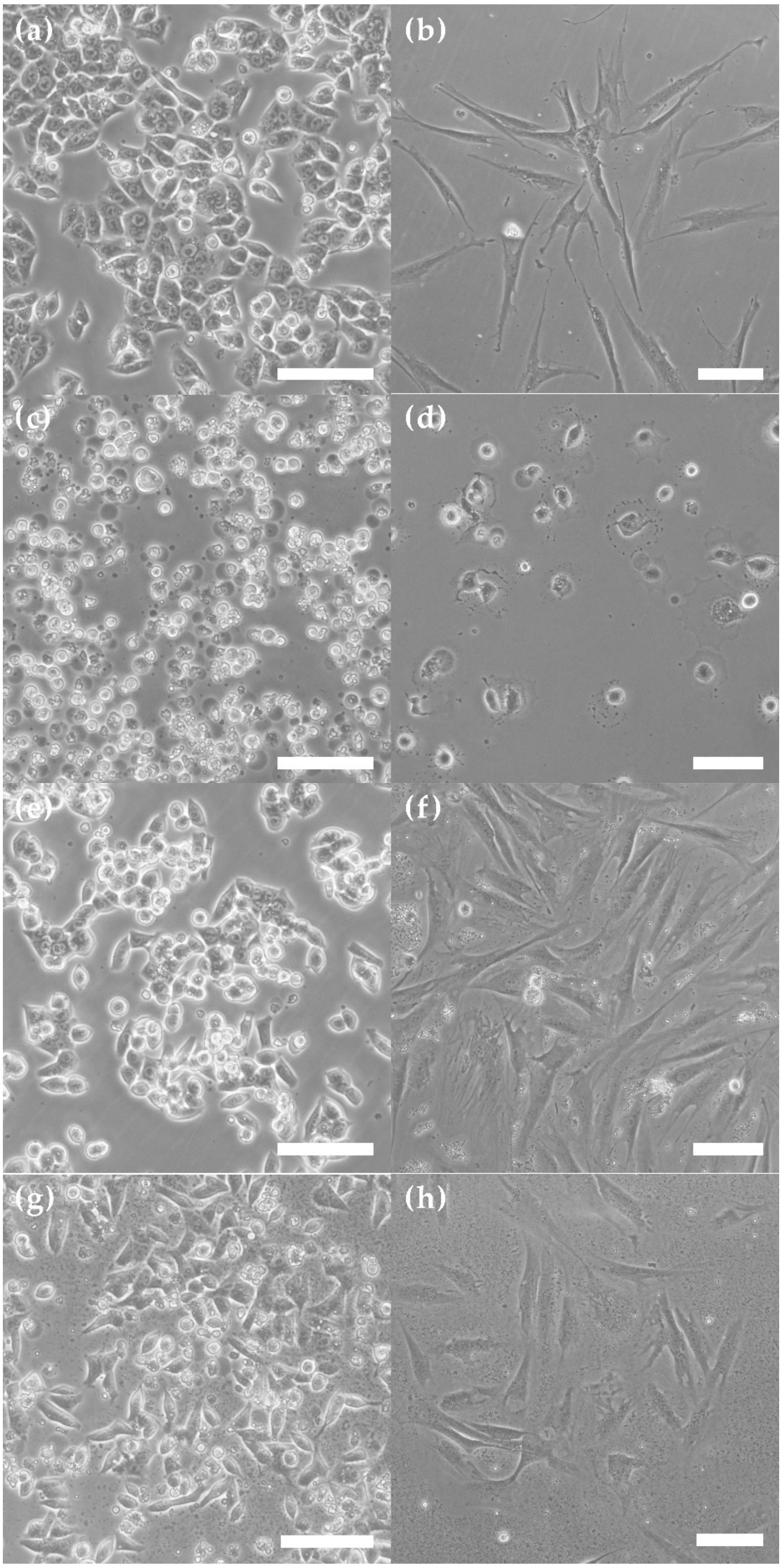

Figure S5. The morphology of human colon carcinoma cell line (HCT 116) (a, c, e, and g) and primary human dermal fibroblasts (HDF) (b, d, f, and h) cultivated in the presence of gels for 3.5, 24 and 48 h: control cells (a, b), and cells cultured with SA:CEC 1:5 (c, d), GA:CEC 1:50 (e, f), MbSA:CEC 1:50 (g, h). Specimens were examined in a CKX41 inverted microscope (Olympus, Japan) equipped with phase-contrast optics and imaged with an Axiocam 105 color digital camera (Carl Zeiss, Germany) controlled by ZEN 2 blue edition software (Carl Zeiss). Scale bar – 100 μm.

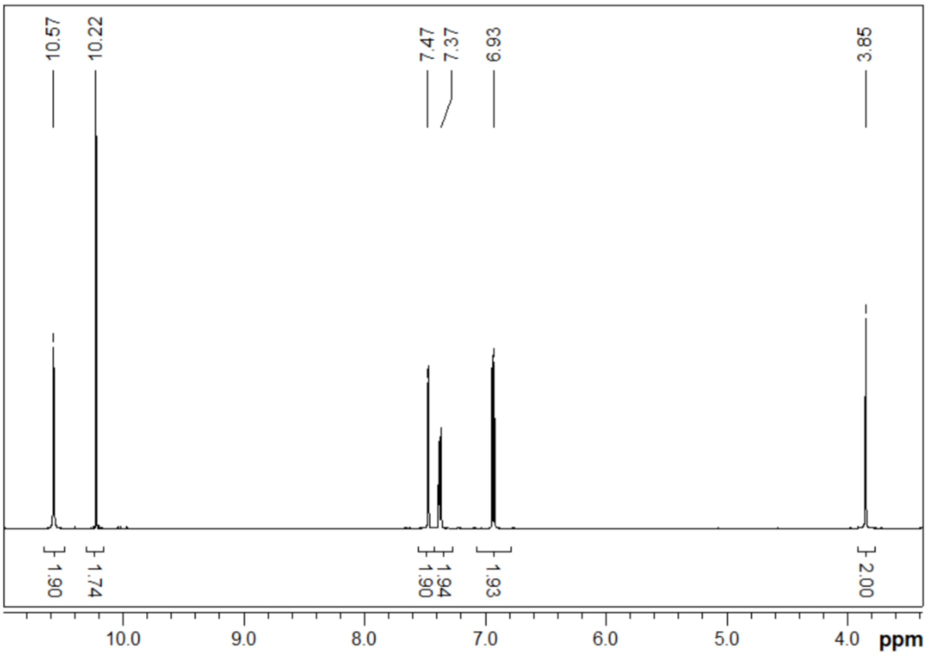

Figure S6. 400 MHz ^{1}H NMR spectra (DMSO d_6) of methylenebis(salicylaldehyde)

Table S1. Rheological parameters of the CEC-hydrogels at angular frequency of 11.2 Hz (full mechanical spectra are shown in Figs. 1 and 2).

Hydrogel*	**T(°C)**	**Storage modulus (G′, Pa)**	**Loss modulus (G″, Pa)**	**Complex viscosity (Pa·s)**
SA:CEC 1:5 1 h	25	166	13.4	14.9
SA:CEC 1:5 72 h	25	1020	47.4	91
SA:CH-HMW 1:5 72 h	25	24.9	30.8	3.54
MbSA:CH-HMW 1:50 72 h	25	180	26.9	16.2
MbSA:CEC 1:10 72 h	25	1730	659	166
MbSA:CEC 1:30 72 h	25	1110	459	107
MbSA:CEC 1:30 72 h	37	1570	134	141
MbSA:CEC 1:50 72 h	25	1210	247	110
MbSA:CEC 1:50 72 h	37	1350	9.88	120
MbSA:CEC 1:50 1 h	37	1040	14.3	93.1
MbSA:CEC 1:50 24 h	25	621	62.4	55.8
MbSA:CEC 1:50 24 h	37	1260	180	113
MbSA:CEC 1:50 1 h	25	30	2.15	2.69
GA:CEC 1:50 72 h	25	428	6.29	38.2
GA:CEC 1:30 72 h	25	1120	14.6	100
GA:CEC 1:10 72 h	25	1680	270	152

*- *Notations indicate aldehyde, polymer, aldehyde: polymer molar ratio, and gelation time. CH-HMW is high molecular weight chitosan, CEC is N-(2-carboxyethyl)chitosan, SA is salicylaldehyde, MbSA is methylenebis(salicylaldehyde), GA is glutaraldehyde.*

Table S2. The complete composition of Dulbecco's modified Eagle's medium (#12800017, Gibco, UK, https://www.thermofisher.com/order/catalog/product/12800017)

Components	Molecular Weight	Concentration (mg/L)	mM
Glycine	75.0	30.0	0.4
L-Arginine hydrochloride	211.0	84.0	0.39810428
L-Cystine 2HCl	313.0	63.0	0.20127796
L-Glutamine	146.0	584.0	4.0
L-Histidine hydrochloride-H_2O	210.0	42.0	0.2
L-Isoleucine	131.0	105.0	0.8015267
L-Leucine	131.0	105.0	0.8015267
L-Lysine hydrochloride	183.0	146.0	0.7978142
L-Methionine	149.0	30.0	0.20134228
L-Phenylalanine	165.0	66.0	0.4
L-Serine	105.0	42.0	0.4
L-Threonine	119.0	95.0	0.79831934
L-Tryptophan	204.0	16.0	0.078431375
L-Tyrosine disodium salt dihydrate	261.0	104.0	0.39846742
L-Valine	117.0	94.0	0.8034188
Choline chloride	140.0	4.0	0.028571429
D-Calcium pantothenate	477.0	4.0	0.008385744
Folic Acid	441.0	4.0	0.009070295
Niacinamide	122.0	4.0	0.032786883

Components	Molecular Weight	Concentration (mg/L)	mM
Pyridoxine hydrochloride	206.0	4.0	0.019417476
Riboflavin	376.0	0.4	0.0010638298
Thiamine hydrochloride	337.0	4.0	0.011869436
i-Inositol	180.0	7.2	0.04
Calcium Chloride ($CaCl_2$) (anhyd.)	111.0	200.0	1.8018018
Ferric Nitrate ($Fe(NO_3)_3$"$9H_2O$)	404.0	0.1	2.4752476E-4
Magnesium Sulfate ($MgSO_4$) (anhyd.)	120.0	97.67	0.8139166
Potassium Chloride (KCl)	75.0	400.0	5.3333335
Sodium Chloride (NaCl)	58.0	6400.0	110.344826
Sodium Phosphate monobasic (NaH_2PO_4-H_2O)	138.0	125.0	0.9057971
D-Glucose (Dextrose)	180.0	4500.0	25.0
Phenol Red	376.4	15.0	0.039851222
Sodium Pyruvate	110.0	110.0	1.0
